# Establishment of a Knock-In Mouse Model with the *SLC26A4* c.919-2A>G Mutation and Characterization of Its Pathology

**DOI:** 10.1371/journal.pone.0022150

**Published:** 2011-07-21

**Authors:** Ying-Chang Lu, Chen-Chi Wu, Wen-Sheng Shen, Ting-Hua Yang, Te-Huei Yeh, Pei-Jer Chen, I-Shing Yu, Shu-Wha Lin, Jau-Min Wong, Qing Chang, Xi Lin, Chuan-Jen Hsu

**Affiliations:** 1 Institute of Biomedical Engineering, National Taiwan University, Taipei, Taiwan; 2 Department of Otolaryngology, National Taiwan University Hospital, Taipei, Taiwan; 3 Graduate Institute of Clinical Medicine, College of Medicine, National Taiwan University, Taipei, Taiwan; 4 Department of Medical Genetics, National Taiwan University Hospital, Taipei, Taiwan; 5 Transgenic Mouse Models Core (TMMC), Division of Genomic Medicine, Research Center for Medical Excellence, National Taiwan University, Taipei, Taiwan; 6 Department of Otolaryngology, Emory University School of Medicine, Atlanta, Georgia, United States of America; 7 Department of Otolaryngology, College of Medicine, National Taiwan University, Taipei, Taiwan; The University of Hong Kong, China

## Abstract

Recessive mutations in the *SLC26A4* gene are a common cause of hereditary hearing impairment worldwide. Previous studies have demonstrated that different *SLC26A4* mutations may have different pathogenetic mechanisms. In the present study, we established a knock-in mouse model (i.e., *Slc26a4^tm1Dontuh/tm1Dontuh^* mice) homozygous for the c.919-2A>G mutation, which is a common mutation in East Asians. Mice were then subjected to audiologic assessment, a battery of vestibular evaluations, and inner ear morphological studies. All *Slc26a4^tm1Dontuh/tm1Dontuh^* mice revealed profound hearing loss, whereas 46% mice demonstrated pronounced head tilting and circling behaviors. There was a significant difference in the vestibular performance between wild-type and *Slc26a4^tm1Dontuh/tm1Dontuh^* mice, especially those exhibiting circling behavior. Inner ear morphological examination of *Slc26a4^tm1Dontuh/tm1Dontuh^* mice revealed an enlarged endolymphatic duct, vestibular aqueduct and sac, atrophy of stria vascularis, deformity of otoconia in the vestibular organs, consistent degeneration of cochlear hair cells, and variable degeneration of vestibular hair cells. Audiologic and inner ear morphological features of *Slc26a4^tm1Dontuh/tm1Dontuh^* mice were reminiscent of those observed in humans. These features were also similar to those previously reported in both knock-out *Slc26a4^−/−^* mice and *Slc26a4^loop/loop^* mice with the *Slc26a4* p.S408F mutation, albeit the severity of vestibular hair cell degeneration appeared different among the three mouse strains.

## Introduction

Mutations in the *SLC26A4* (*PDS*, GeneID 5172) gene are the second most frequent cause of human hereditary hearing impairment worldwide, following mutations in the *GJB2* (GeneID 2706) gene [1]. Recessive *SLC26A4* mutations are responsible for both non-syndromic hereditary hearing loss DFNB4 (MIM 600791) [2] and Pendred syndrome (PS, MIM 274600) [3], two disorders commonly encountered in patients with deafness. DFNB4 and PS are characterized by sensorineural hearing impairment accompanied by an enlarged vestibular aqueduct (EVA, MIM 603545) and/or incomplete partition of the cochlea (i.e., Mondini dysplasia), although the latter also includes development of goiter. *SLC26A4* encodes pendrin, an iodide/chloride/bicarbonate transporter expressed in the inner ear, thyroid, and kidney [4], [5], [6], [7].

In recent years, the understanding of the pathogenesis of DFNB4 and PS has been facilitated by various studies performed in the knock-out mouse model *Slc26a4* ^−/−^ [8]. However, the phenotypes observed in *Slc26a4* ^−/−^ mice, such as unanimously profound hearing loss and the absence of thyroid pathology in all littermates, fail to completely reflect those observed in humans with various *SLC26A4* mutations. Clinically, patients with *SLC26A4* mutations have been shown to demonstrate a broad-spectrum of phenotypes, ranging from non-syndromic isolated EVA to full-blown PS with goiter and incomplete partition of the cochlea in addition to EVA. To date, more than 100 *SLC26A4* mutations have been identified (Pendred/BOR Homepage; www.healthcare.uiowa.edu/labs/pendredandbor). It was reported that patients with different *SLC26A4* genotypes were correlated to distinct clinical phenotypes, with PS patients more likely to have 2 *SLC26A4* mutant alleles than those with non-syndromic EVA [9], [10]. Furthermore, cell line studies have demonstrated that certain *SLC26A4* mutations only impair the function of pendrin partially, instead of completely ablating the protein function [11], [12]. These lines of evidence indicate that the pathogenetic mechanisms of *SLC26A4* mutations in humans to some extent differ from those mechanisms present in the *Slc26a4* ^−/−^ mouse model, in which exon 8 of the *Slc26a4* gene (GeneID 23985) is knocked out.

To elucidate the discrepancies in phenotypes observed between humans and *Slc26a4* ^−/−^ mice, it might be necessary to create knock-in mouse models that harbor the corresponding human *SLC26A4* mutations. Accordingly, the primary purpose of the present study was to establish a knock-in mouse model homozygous for the c.919A>G mutation, which is the most prevalent *SLC26A4* mutation in Chinese [13], [14] individuals, and the second most prevalent mutation in Japanese [15] and Koreans [16]. We also characterized the associated audiological and vestibular phenotypes as well as the inner ear pathology in this model.

## Results

### Pathogenetic mechanisms of the c.919-2A>G mutation

**Figure 1:**
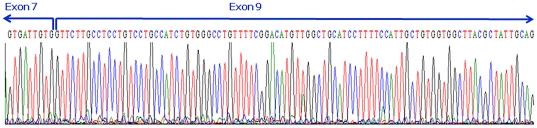
The *Slc26a4* transcripts from the allele with the c.919-2A>G mutation lead to a complete omission of exon 8 during the splicing process in *Slc26a4^tm1Dontuh/tm1Dontuh^* mice.

**Figure 2:**
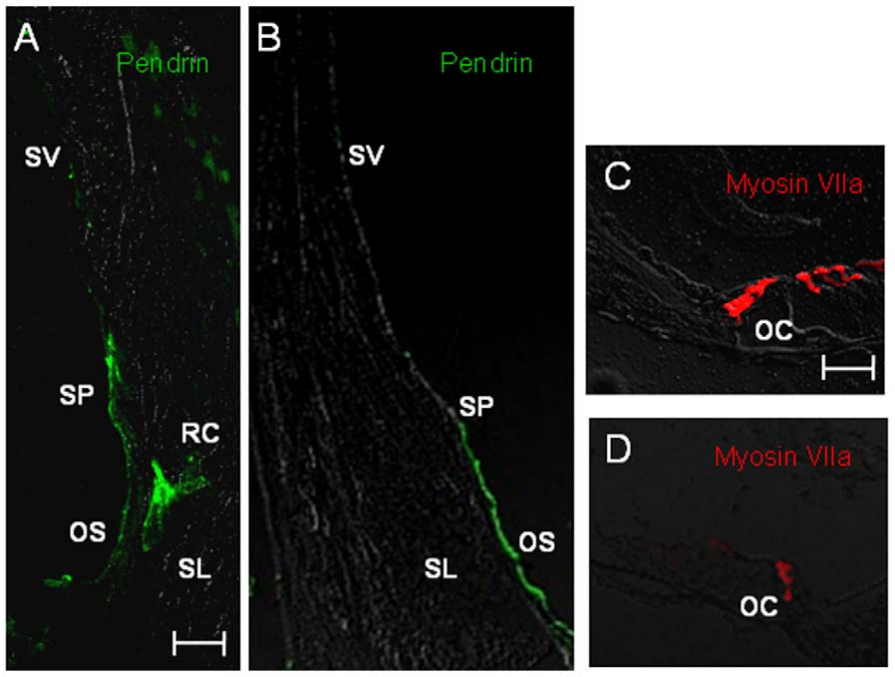
Immunolocalization of pendrin in *Slc26a4^+/+^* mice (A) and *Slc26a4^tm1Dontuh/tm1Dontuh^* mice (B). The expression of pendrin (stained in green) in spiral prominence and outer sulcus epithelial cells was not affected by the c.919-2A>G mutation. Degeneration of cochlear hair cells in *Slc26a4^tm1Dontuh/tm1Dontuh^* mice was revealed by fluorescence confocal microscopy (D). (OC, organ of Corti; OS, outer sulcus epithelial cells; RC, root cells; SP, spiral prominence; SL, spiral ligament; SV, stria vascularis). Bar = 50 µm.

In our previous study, the c.919-2A>G mutation was expected to lead to aberrant splicing, based on a computer-assisted analysis by the Neural Network at the Berkeley Drosophila Genome Project (BDGP; www.fruitfly.org/seq_tools/splice.html; splice score = 0 compared to 0.93 of the wild-type sequence [17]. In transformed lymphocytes, sequencing of the RT-PCR products revealed that *SLC26A4* transcripts from the allele with c.919-2A>G lose the entire exon 8, resulting in fusion of exons 7 and 9 [18]. In the present study, we confirmed that c.919-2A>G indeed led to a complete omission of exon 8 during the splicing process in inner ear and kidney (Fig. 1). The frameshift generated a new stop codon at position 348, resulting in prematurely truncated pendrin (347 amino acids). However, pendrin localization in the inner ear was not affected by the c.919-2A>G mutation, as shown by immunofluorescence studies (Fig. 2).

### Audiological and vestibular phenotypes

**Figure 3:**
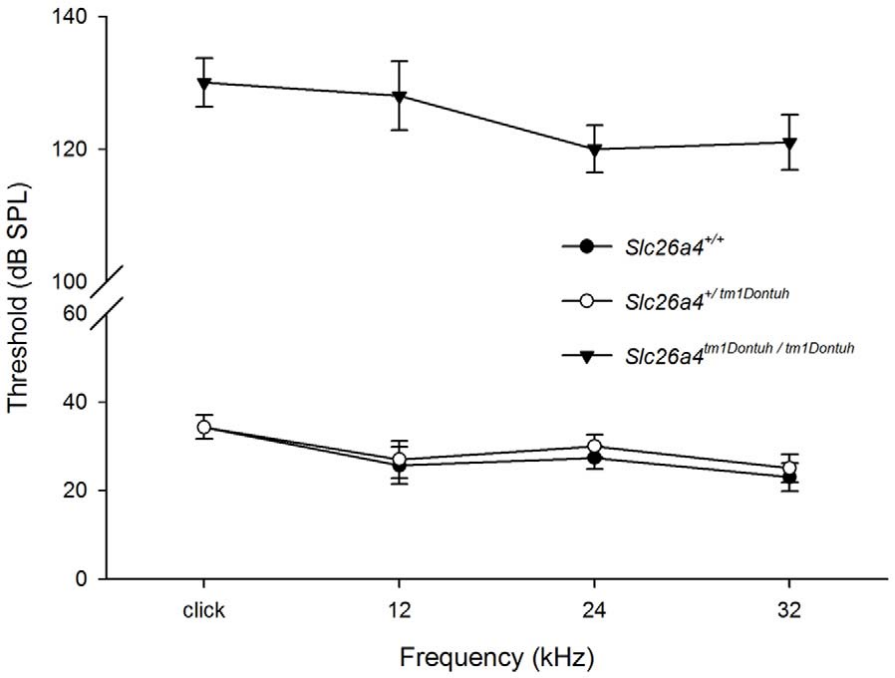
Audiological phenotypes of *Slc26a4^tm1Dontuh/tm1Dontuh^* mice. All *Slc26a4^tm1Dontuh/tm1Dontuh^* mice (n = 15) revealed profound hearing loss (>120 dB SPL) according to ABR at all frequencies, whereas all *Slc26a4^+/tm1Dontuh^* mice (n = 15) and all *Slc26a4^+/+^* mice (n = 15) revealed normal hearing.

A total of 45 mice (P35–P42), including 15 *Slc26a4^+/+^* mice, 15 *Slc26a4^+/tm1Dontuh^* mice and 15 *Slc26a4^tm1Dontuh/tm1Dontuh^* mice, were subjected to audiological evaluations. All *Slc26a4^tm1Dontuh/tm1Dontuh^* mice revealed profound hearing loss (>120 dB SPL) on ABR in all frequencies, whereas all *Slc26a4^+/tm1Dontuh^* and *Slc26a4^+/+^* mice revealed normal hearing (Fig. 3). These results indicate that the c.919-2A>G mutation of *Slc26a4* was inherited in an autosomal recessive manner in mice, as in humans. The audiological characteristics were observed until mice reached 12 months in age. We found no obvious change in hearing ability of these mice.

None of the *Slc26a4^+/tm1Dontuh^* or *Slc26a4^+/+^* mice revealed vestibular deficits, whereas 46% (23/50) of *Slc26a4^tm1Dontuh/tm1Dontuh^* mice demonstrated pronounced head-tilting and circling behaviors before reaching 3 weeks of age. The phenotype of circling behavior did not change until 12 months. A total of 60 mice, including 15 *Slc26a4^+/+^* mice, 15 *Slc26a4^+/tm1Dontuh^* mice, 15 *Slc26a4^tm1Dontuh/tm1Dontuh^* mice that did not exhibit circling and 15 *Slc26a4^tm1Dontuh/tm1Dontuh^* mice that did exhibit circling, were subjected to vestibular evaluations (Table 1). In general, *Slc26a4^tm1Dontuh/tm1Dontuh^* mice revealed impaired balancing ability compared to wild-type mice. The presence of circling behaviors was in complete accord with the presence of head-tilting in *Slc26a4^tm1Dontuh/tm1Dontuh^* mice. Furthermore, circling mice were associated with a significantly poorer performance on reaching, swimming, gripping, and rotorod tests. *Slc26a4^tm1Dontuh/tm1Dontuh^* mice without circling behaviors showed only a minor degree of vestibular dysfunction, yet their performance on the rotorod test was also poorer than wild-type mice. We also noted a significant difference in vestibular features, including reaching, swimming, gripping, and balancing on the rotorod, between *Slc26a4^tm1Dontuh/tm1Dontuh^* mice that exhibited circling and those that did not (p<0.05, in all cases).

**Table 1:** Comparison of vestibular features according to the genotypes and the circling behavior.

	*Slc26a4^+/+^*	*Slc26a4^+/tm1Dontuh^*	*Slc26a4^tm1Dontuh/tm1Dontuh^*	*Slc26a4^tm1Dontuh/tm1Dontuh^*
	(n = 15)	(n = 15)	(Non-circling)	(Circling)
			(n = 15)	(n = 15)
Head tilting* (n, abnormal/total)	0/15	0/15	0/15†,	15/15‡
Reaching§ (n, abnormal/total)	0/15	0/15	4/15†	11/15‡
Swimming§ (n, abnormal/total)	0/15	0/15	2/15†	11/15‡
Gripping§ (n, abnormal/total)	0/15	0/15	3/15†	13/15‡
Time on rod§ (sec)	164.7±13.9	159.3±18.1	66.3±8.9‡	4.0±1.6‡

*Observed at 3 weeks.

§ Tested at 8 weeks.

† *p*>0.05 as compared to wild type.

‡ *p*<0.01 as compared to wild type.

### Inner ear morphology: endolymphatic duct, vestibular aqueduct and sac

**Figure 4:**
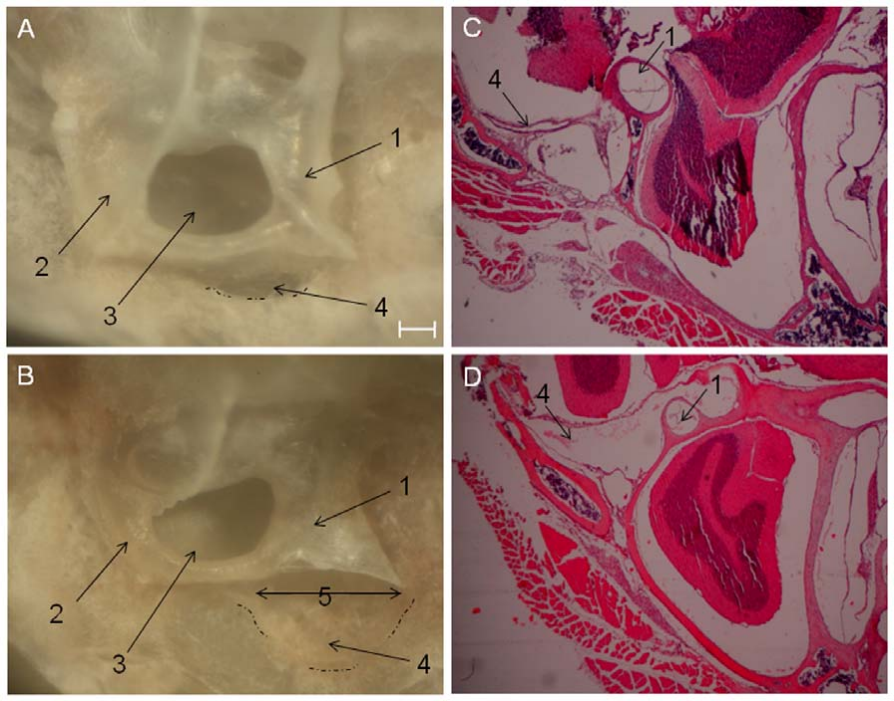
Structural aberrations of the vestibular aqueduct and endolymphatic sac in mice. The vestibular aqueduct and endolymphatic sac in the *Slc26a4^tm1Dontuh/tm1Dontuh^* mice were enlarged (B & D) compared to those in the *Slc26a4^+/+^* mice (A & C). (1, posterior semicircular canal; 2, superior semicircular canal; 3, subarcuate fossa; 4, fovea for endolymphatic sac (dot-dash line); 5, external orifice of vestibular aqueduct. Bar = 100 µm.

When observed with the naked eye, the orifice of vestibular aqueduct and fovea of endolymphatic sac were noticeably enlarged in *Slc26a4^tm1Dontuh/tm1Dontuh^* mice (Fig. 4B, as indicated by arrows and dot-dash line), as compared to those in *Slc26a4^+/+^* mice (Fig. 4A). This structural aberrance was also identified with histological studies by H&E staining (Fig. 4D vs. Fig. 4C).

### Inner ear morphology: cochlea

**Figure 5:**
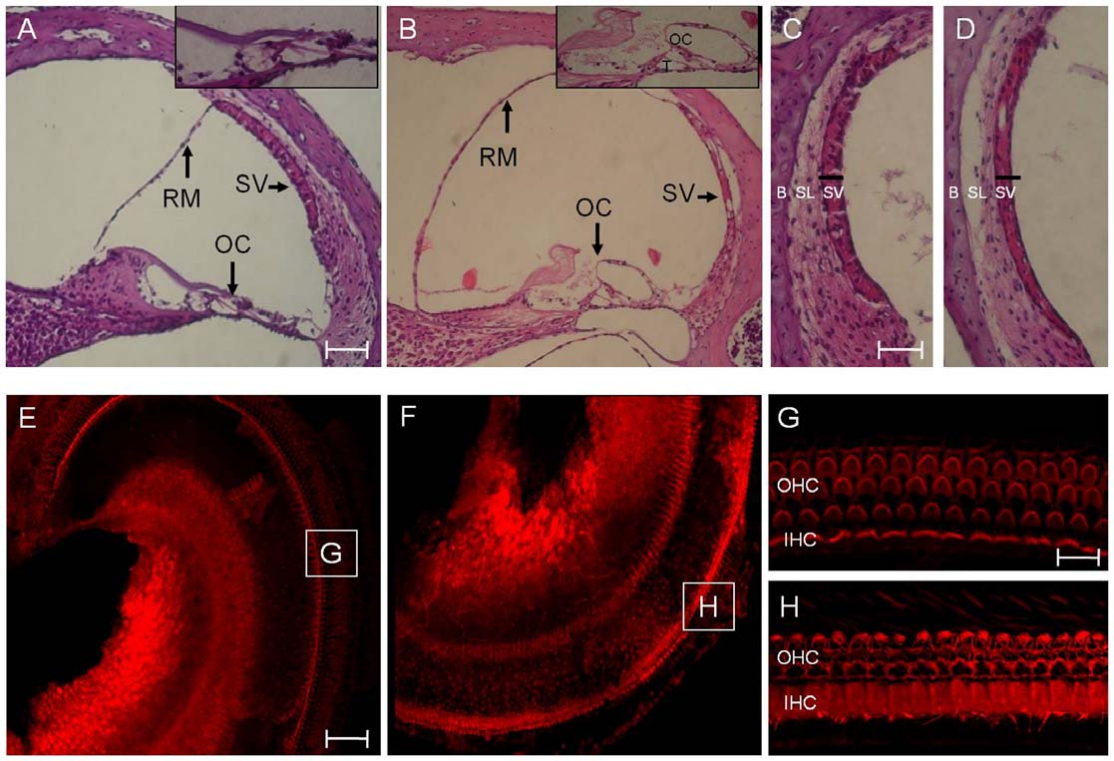
Comparison of cochlear morphology between *Slc26a4^+/+^* mice (A, C, E & G) and *Slc26a4^tm1Dontuh/tm1Dontuh^* mice (B, D, F & H). Microscopic examination of the cochlear duct revealed severe endolymphatic hydrops with dilatation of scala media, degeneration of hair cells (B) and a significant atrophy of the stria vascularis (D) in *Slc26a4^tm1Dontuh/tm1Dontuh^* mice. Degeneration of cochlear hair cells in *Slc26a4^tm1Dontuh/tm1Dontuh^* mice was also revealed by fluorescence confocal microscopy (H). G, H: magnification of boxes G and H from figures E and F, respectively. B, bone; IHC, inner hair cells; OC, organ of Corti; OHC, outer hair cells; RM, Reissner's membrane; SL, spiral ligament; SV, stria vascularis; T, tunnel of Corti. Bar = 150 µm (A & B), 50 µm (C, D, E & F), and 10 µm (G & H).

**Figure 6:**
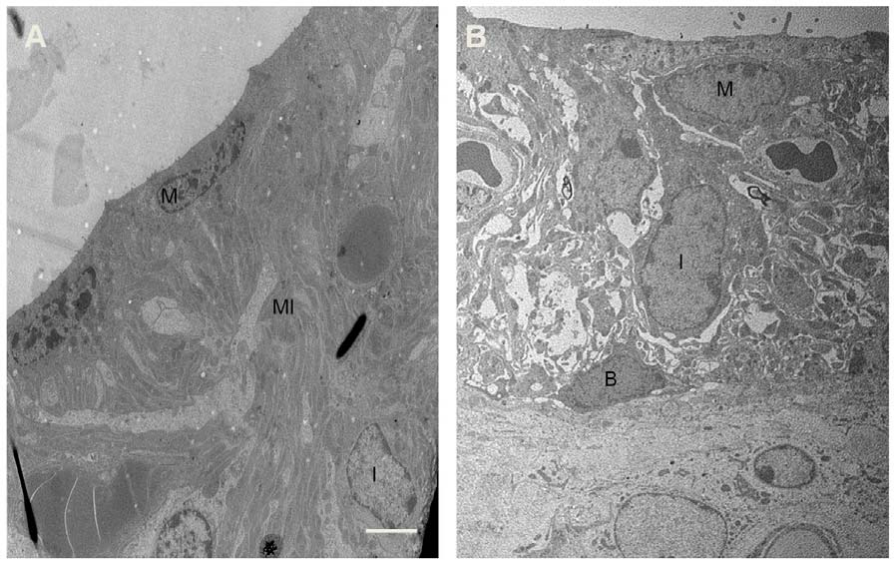
Transmission electron micrographs of the stria vascularis in *Slc26a4^+/+^* mice (A) and *Slc26a4^tm1Dontuh/tm1Dontuh^* mice (B). The most significant change in stria vascularis in *Slc26a4^tm1Dontuh/tm1Dontuh^* mice was diminished membrane invaginations of marginal cell with vacuolization changes. B, basal cell; I, intermediate cell; M, marginal cell; MI, membrane invaginations. Bar = 5 µm.

Examination of the cochlear duct from 4-week-old mice revealed severe endolymphatic hydrops with dilatation of scala media in *Slc26a4^tm1Dontuh/tm1Dontuh^* mice (Fig. 5B), compared to *Slc26a4^+/+^* mice. A higher magnification view also showed degeneration of hair cells (Fig. 5B) and a significant atrophy of the stria vascularis (Fig. 5D) in all *Slc26a4^tm1Dontuh/tm1Dontuh^* mice by 6 weeks. Degeneration of inner and outer hair cells in the organ of Corti could also be confirmed by fluorescence confocal microscopy (Fig. 5H). Further investigation of the stria vascularis by TEM revealed that the most significant change was the diminished membrane invaginations of marginal cell with vacuolization changes or interstitial edema (Fig. 6).

### Inner ear morphology: vestibular system

**Figure 7:**
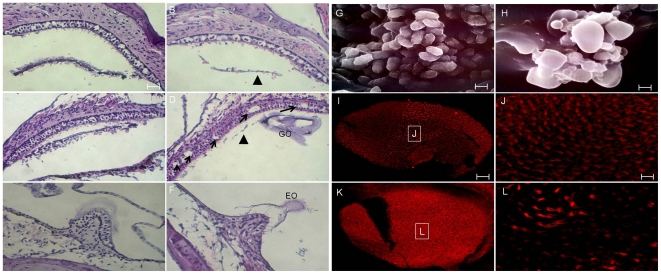
Comparison of vestibular morphology between *Slc26a4^+/+^* mice (A, saccule; C, utricle; E, semicircular canal; G, utricular macula) and *Slc26a4^tm1Dontuh/tm1Dontuh^* mice (B, saccule; D, utricle; F, semicircular canal; I, utricular macula). *Slc26a4^tm1Dontuh/tm1Dontuh^* mice revealed a decreased amount of otoconia in the saccule and utricle (B & D, arrowhead), formation of “giant otoconia” in the utricle (D & H), ectopic otoconia in the semicircular canal (F), as well as a wide spectrum of vestibular hair cell loss and degeneration (arrows) at 8 weeks of age. Scanning electron microscopy reveals the markedly enlarged otoconia at saccule and utricle in *Slc26a4^tm1Dontuh/tm1Dontuh^* mice (H) as compared with wild-type (G). Degeneration of vestibular hair cells in *Slc26a4^tm1Dontuh/tm1Dontuh^* mice was also revealed by fluorescence confocal microscopy (L). J, L: magnification of boxes J and L from figures I and K, respectively. EO, ectopic otoconia; GO, giant otoconia. Bar = 50 µm (A–F, I & K), and 10 µm (G, H, J & L).

Morphological changes of the vestibular organs in *Slc26a4^tm1Dontuh/tm1Dontuh^* mice included deformity of otoconia and degeneration of the sensory epithelium. To be more specific, deformity included a decreased amount of otoconia present in the saccule and utricle (Fig. 7A), formation of “giant otoconia” in the saccule and utricle (Fig. 7D) and ectopic distribution of otoconia into the semicircular canal (Fig. 7F). Further investigation of the otoconia by SEM revealed that the giant otoconia in the saccule and utricle in *Slc26a4^tm1Dontuh/tm1Dontuh^* mice (Fig. 7H) as compared with wild-type mice (Fig. 7G). Furthermore, in contrast to the consistent degeneration of cochlear hair cells seen in 6-weeks-old *Slc26a4^tm1Dontuh/tm1Dontuh^* mice, 8-week-old mice revealed a wide spectrum of hair cell loss and degeneration in the saccule (Fig. 7B), utricle (Fig. 7D & Fig. 7J) and semicircular canals (Fig. 7F). Of note, hair cells in the utricle appeared to be more affected than those in the saccule and semicircular canals. The morphological findings in the vestibular organs, including the abnormality of otoconia (defined by decreased amount of otoconia in the saccule and utricle, formation of giant otoconia in the utricle and ectopic distribution of otoconia in the semicircular canal), and the degeneration of hair cells (measured by calculating the number of areas of hair cell loss in 5 random sections per mouse), were then correlated to the vestibular phenotype of circling. As shown in Table 2, we detected no significant difference in the abnormality of otoconia between those mice that exhibited circling and those mice that did not, but the degeneration and loss of utricular hair cells were more significant in *Slc26a4^tm1Dontuh/tm1Dontuh^* mice that exhibited circling.

**Table 2:** Comparison of vestibular morphological findings according to the vestibular phenotype of circling.

	*Slc26a4^tm1Dontuh/tm1Dontuh^*	*Slc26a4^tm1Dontuh/tm1Dontuh^*	*p*-value*
	(Circling)	(Non-circling)	
**Saccule**			
Areas of hair cell loss† (n of areas/animal)	0.6±0.9	0.4±0.9	*p*>0.05
Otoconia (n of animal, abnormal‡/total)	5/5	5/5	*p*>0.05
**Utricle**			
Areas of hair cell loss (n of areas/animal)	9.6±5.9	1.6±3.0	*p*<0.05
Otoconia (n of animal, abnormal‡/total)	5/5	5/5	*p*>0.05
**Semicircular canal**			
Areas of hair cell loss (n of areas/animal)	2.4±2.2	1.6±2.2	*p*>0.05
Ectopic otoconia (n of animal, abnormal‡/total)	4/5	5/5	*p*>0.05

*Fisher's exact test for categorical variables and Student's *t*-test for continuous variables.

† Measured by calculating the number of areas of hair cell loss in 5 random sections pre mouse.

‡ Defined by decreased amount of otoconia in the saccule and utricle, formation of giant otoconia in the utricle and ectopic distribution of otoconia in the semicircular canal.

### Thyroid and renal profiles

Goiter was not observed in *Slc26a4^tm1Dontuh/tm1Dontuh^* mice (n = 30) until 12 months in age. Total T_4_ in 2-month-old (n = 10) and 6-month-old (n = 10) *Slc26a4^tm1Dontuh/tm1Dontuh^* mice were 3.34±0.14 µg/dl and 3.03±0.16 µg/dl, respectively; whereas total T_4_ in 2-month-old (n = 10) and 6-month-old (n = 10) wild type mice were 3.46±0.20 µg/dl and 3.08±0.20 µg/dl, respectively, showing no difference between *Slc26a4^tm1Dontuh/tm1Dontuh^* and wild type mice. Similarly, *Slc26a4^tm1Dontuh/tm1Dontuh^* mice revealed normal renal functions up to 6 months. Blood urea nitrogen (BUN) in 2-month-old (n = 10) and 6-month-old (n = 10) *Slc26a4^tm1Dontuh/tm1Dontuh^* mice were 31.4±4.14 mg/dl and 33.3±5.18 mg/dl, respectively; whereas BUN in 2-month-old (n = 10) and 6-month-old (n = 10) wild type mice were 30.2±5.26 mg/dl and 32.4±4.20 mg/dl, respectively. Creatinine (CREA) in 2-month-old (n = 10) and 6-month-old (n = 10) *Slc26a4^tm1Dontuh/tm1Dontuh^* mice were 0.19±0.06 mg/dl and 0.21±0.04 mg/dl, respectively; whereas CREA in 2-month-old (n = 10) and 6-month-old (n = 10) wild type mice were 0.21±0.06 mg/dl and 0.22±0.02 mg/dl, respectively. There was no significant difference in BUN and CREA between *Slc26a4^tm1Dontuh/tm1Dontuh^* and wild type mice up to 6 months.

## Discussion

In this study, we generated a knock-in mouse model, denoted as *Slc26a4^tm1Dontuh/tm1Dontuh^* mice, which segregates a common deafness-associated mutation, c.919-2A>G, in humans. Phenotypic characterization of *Slc26a4^tm1Dontuh/tm1Dontuh^* mice revealed they had profound hearing impairment, impaired balancing ability, an enlarged vestibular aqueduct, degeneration of cochlear and vestibular hair cells and atrophy of stria vascularis. To our knowledge, there are only two other mouse mutants in the literature that harbor *Slc26a4* mutations: the knock-out *Slc26a4^−/−^* mouse model [8] and the *Slc26a4^loop/loop^* mouse, which segregates the *Slc26a4* p.S408F mutation generated by the ENU mutagenesis project [19]. From the pathogenetic perspective, *Slc26a4^−/−^* and *Slc26a4^tm1Dontuh/tm1Dontuh^* mice share a loss of exon 8 of the *Slc26a4* transcript, resulting in a truncated protein; whereas the p.S408F mutation of *Slc26a4^loop/loop^* mice leads to an aberrant protein. The audiological, vestibular, and inner ear morphological findings of *Slc26a4^−/−^*, *Slc26a4^loop/loop^* and *Slc26a4^tm1Dontuh/tm1Dontuh^* mice are summarized in Table 3. Homozygous mice of all three strains revealed profound hearing loss, which indicates that the c.919-2A>G mutation of *Slc26a4* is inherited in an autosomal recessive manner. In contrast, the severity of vestibular phenotypes appeared variable: some mice showed marked vestibular deficits such as head-tilting and circling behaviors, whereas the others remained relatively normal.

**Table 3:** Comparison of phenotypes among mouse strains segregating different slc26a4 mutations.

	*Slc26a4^−/−^*	*Slc26a4^loop/loop^*	*Slc26a4^tm1Dontuh/tm1Dontuh^*
Audiological phenotypes	Profound hearing loss	Profound hearing loss	Profound hearing loss
	(>100 dB SPL)	(>100 dB SPL)	(>120 dB SPL)
Vestibular phenotypes	Vestibular deficits, including head-tilting, head-bobbing unsteadiness, circling and abnormal reaching response in ∼50% mice.	Variable vestibular deficits, including unsteady gait, circling, absence of reaching response and tilted body. Proportion of mice with vestibular deficits not reported.	46% of mice with head-tilting and circling. Poorer vestibular function in *Slc26a4^tm1Dontuh/tm1Dontuh^* mice, esp. in those with circling.
Inner Ear Morphology			
Vestibular aqueduct	Enlarged	ND	Enlarged
Scala media	Enlarged	ND	Enlarged
Stria vascularis	Atrophic	ND	Atrophic
Cochlear hair cells	Severe degeneration of inner and outer hair cells by P30.	ND	Severe degeneration of inner and outer hair cells at 6 wks.
Vestibular hair cells	Severe degeneration of vestibular hair cells by P30.	Normal morphology of vestibular hair cells at 2 m.	Loss and degeneration of utricular hair cells correlated to the vestibular phenotypes
Otoconia	Almost complete absence of otoconia with occasional presence of giant otoconia.	Giant otoconia in the utricle from P0 to 10 m; gradual change in otoconia composition to calcium oxalate in the saccule from P0 to 10 m; ectopic otoconia in the semicircular canal.	Decreased amount of otoconia in the saccule and utricle, formation of giant otoconia in the saccule and utricle, and ectopic distribution of otoconia into the semicircular canal

ND, not described.

* *Slc26a4^loop/loop^*: mice homozygous for the p.S408F mutation.

It has been reported that mice with different *Slc26a4* mutations revealed different degrees of vestibular degeneration. In particular, the morphology of utricular hair cells in 2-month-old *Slc26a4^loop/loop^* mice was found to be normal [19], whereas *Slc26a4^−/−^* mice show severe utricular hair cell degeneration by the age of 1 month [8]. As demonstrated in the present study, *Slc26a4^tm1Dontuh/tm1Dontuh^* mice seem to have an intermediate grade of morphological changes, with the severity of vestibular hair cell degeneration related to the presence of vestibular deficits. These findings suggest that a genotype-phenotype correlation can be deduced among mice with different *Slc26a4* mutations. In humans, it has been reported that PS patients are more likely to have 2 *SLC26A4* mutant alleles than those with non-syndromic EVA [9], [10]. Moreover, the number of *SLC26A4* mutant alleles is also significantly correlated to the severity of hearing loss in individuals with EVA [20]. However, because of its diverse mutation spectrum, it is difficult to delineate the phenotypes associated with a specific *SLC26A4* mutation in humans. Consequently, determination of the pathogenetic role of each specific *SLC26A4* mutation largely relies on functional studies performed in cell lines [11], [12], [21]. The observation that mice with distinct *Slc26a4* mutations reveal different phenotypes indicates that transgenic mice may serve as an appropriate, direct model to investigate corresponding *SLC26A4* mutations in humans.

Recent studies have provided excellent insights into how *SLC26A4* mutations lead to hearing impairment. It has been proposed that *Slc26a4* ^−/−^ mice develop deafness via several mechanisms. First, loss of cochlear HCO_3_^−^ secretion results in the acidification of endolymph, which consequently increases Ca^2+^ concentration in the endolymph by inhibiting Ca^2+^ reabsorption [22], [23] leads to free radical stress-mediated loss of *Kcnj10* protein expression in stria vascularis, ultimately abolishing the production of endocochlear potential [24]. Second, local hypothyroidism in the cochlea may contribute to delays in cochlear development, which can lead to the inability to develop hearing [25]. In addition to the degeneration of cochlear hair cells, *Slc26a4* ^−/−^ and *Slc26a4^tm1Dontuh/tm1Dontuh^* mice show atrophy of stria vascularis, which is uncommon in other mouse models for hereditary hearing impairment. According to a previous study, atrophy of stria vascularis may cause degeneration of hair cells by alteration in endolymph composition [26]. Since *Slc26a4* mutant mice revealed normal cochlear hair cells at a younger age (P7) (Fig. S1), it is conceivable that the pathology in the stria vascularis plays a pivotal role in the later degeneration of cochlear hair cells associated with *SLC26A4* mutations. In the present study, we investigated the morphology of stria vascularis using TEM, and identified that the most significant change in *Slc26a4^tm1Dontuh/tm1Dontuh^* mice was the disappearance of marginal cell invaginations with vacuolization changes. Interestingly, ablation of primary and secondary processes of marginal cells with normal appearing intermediate cells and basal cells has been documented as an end-stage strial change in presbycusic gerbils [27]. It is suggested that the basal part of marginal cells endure a greater exposure to free radicals by virtue of harboring abundant mitochondria, thus being more vulnerable to aging-related injury [27]. The similar morphological strial changes seen in *Slc26a4^tm1Dontuh/tm1Dontuh^* mice support the previous hypothesis that free radicals are crucial for the development of stria vascularis atrophy and the ensuing degeneration of cochlear hair cells.

In comparison to hearing loss, less is known concerning the vestibular dysfunction associated with *SLC26A4* mutations, despite the fact that as many as 70% of patients with *SLC26A4* mutations suffer from episodes of vertigo [28], [29]. In their pioneer study, Everett *et al.* observed giant otoconia at utricle and saccule in *Slc26a4* ^−/−^ mice [30]. Dror *et al.* investigated the mineral composition and morphology of otoconia in *Slc26a4^loop/loop^* mice, and identified abnormal giant carbonate minerals in the utricle of mice at all ages, a gradual change in mineral composition leading to the formation of calcium oxalate in the saccule in adult mice, as well as ectopic giant otoconia in semicircular canals. The authors postulated that the variability of the defective vestibular behavior in *Slc26a4^loop/loop^* mice, ranging from very mild to severe, can be partially explained by the variable position of dislocated mineralized bodies [19]. In the present study, we did not detect a correlation between abnormal otoconia and the presence of circling behavior in *Slc26a4^tm1Dontuh/tm1Dontuh^* mice, but we noted an association between circling behavior and the degeneration of utricular hair cells (Table 2). The underlying factors leading to the degeneration, nevertheless, remain to be clarified.

Although *Slc26a4^−/−^*, *Slc26a4^loop/loop^*, and *Slc26a4^tm1Dontuh/tm1Dontuh^* mice revealed phenotypes reminiscent of those observed in humans, there are some discrepancies that limit direct extrapolation of these results from animal studies into clinical medicine. First, the hearing impairment was profound in these mice, although patients with *SLC26A4* mutations demonstrate a wide variety in audiological features. Some patients exhibit a milder hearing impairment that deteriorates with time, either progressively or after attacks of fluctuating hearing loss [31], [32]. Second, a significant proportion of mutant mice revealed severely impaired vestibular function, but patients with *SLC26A4* mutations usually do not complain of vestibular symptoms, except during attacks of vertigo. Accordingly, further modifications of these mutant mice are necessary to better simulate phenotypes in humans. We submit two possible methods that may alleviate the phenotypes of mice. The first approach includes establishment of a knock-in mouse model with a missense *Slc26a4* mutation other than p.S408F, instead of a mutation that leads to a truncated protein, might result in more minor phenotypes. It has been reported previously that truncating mutations may be associated with a more severe phenotype in certain types of genetic hearing impairments [33]. Alternatively, conditional knock-in mice models that modulate the expression of the *Slc26a4* gene after birth might be helpful in generating animals that are not congenitally deaf.

## Materials and Methods

### Construction of *Slc26a4^tm1Dontuh/tm1Dontuh^* knock-in mice

The mutation gene-targeting vector was constructed using a recombineering approach previously developed by Dr. Copeland's group [34], [35]. From the bMQ323G13 BAC clone (Sanger institute, UK), which carried the entire mouse *Slc26a4* locus, we subcloned a 12.47-kb fragment spanning introns 3 to 10 of *Slc26a4* into the PL253 plasmid (Fig. S2). The subcloned genomic 12.47-kb region was modified in a subsequent targeting round by inserting the neomycin (*neo*) cassette from the PL451 plasmid and creating the c.919-2A>G mutation in intron 7. The conditional targeting vector was then linearized by *Not*I digestion and electroporated into R1 embryonic stem (ES) cells. G418 (240 µg/ml) and Ganciclovir (2 µM) double-resistant clones were analyzed by Southern blot hybridization. The retained *neo* cassette flanked by *FRT* sites was excised *in vivo* by transfecting the targeted clone with plasmid transiently expressing the FLPe recombinase. Established ES clones were then identified by polymerase chain reaction (PCR) screening and subsequently injected into C57BL/6 blastocysts to produce chimeras. Chimeric males were bred with C57BL/6 females to produce *Slc26a4^+/tm1Dontuh^* mice. *Slc26a4^+/tm1Dontuh^* mice (F1) were mated with each other to produce *Slc26a4^+/+^*, *Slc26a4^+/tm1Dontuh^* and *Slc26a4^tm1Dontuh/tm1Dontuh^* mice littermates (F2), and then *Slc26a4^tm1Dontuh/tm1Dontuh^* mice were maintained in C57BL/6 background. All animal experiments were carried out in accordance with animal welfare guidelines and approved by the Institutional Animal Care and Use Committee (IACUC) of National Taiwan University College of Medicine (approval no. 20070077)

### Determination of the pathogenesis of the c.919-2A>G mutation

The pathogenetic mechanisms of the c.919-2A>G mutation were determined by investigating both the mRNA product of mice with this mutation and the consequences of the mutation on pendrin expression. To examine the mRNA product, we purified kidney and inner ear mRNA from both *Slc26a4^+/+^* and *Slc26a4^tm1Dontuh/tm1Dontuh^* mice using Trizol reagent (Invitrogen Carlsbad, CA, USA) according to the manufacturer's instructions. Subsequently, 2 µg of total RNA was reverse-transcribed with a Transcription High Fidelity cDNA Synthesis Kit(RocheDiagnostics Gmbh, Mannheim, Germany), and reverse transcription-PCR (RT-PCR) was carried out using the forward primer 5′-ATCGTGCTCAATGTTTCAACC-3′ and the reverse primer 5′-ATCCAGAGAAGACGTTGCTTATCC-3′. The PCR product length of the wild-type mRNA was determined to be 478 bp encompassing exon 6 (partially) to exon 10 (partially).

For pendrin expression experiments, we prepared tissue sections (5 µm) from the inner ears of *Slc26a4^+/+^* and *Slc26a4^tm1Dontuh/tm1Dontuh^* mice. Tissue sections mounted on silane-coated glass slides were then deparaffinized in xylene and rehydrated via graded ratios of ethanol to water. After antigen heat retrieval (500 W microwave oven, in 10 mm citric buffer, pH 6.0, for 20 min), slides were incubated overnight at 4°C with primary antibodies in PBST (phosphate buffer saline & Tween) [rabbit anti-pendrin, 1∶100 (H195); mouse anti-Myosin VIIa, 1∶100 (C-5) (Santa Cruz Biotechnology, Santa Cruz, CA)]. Slides were then washed and incubated for 1 h at 25°C with appropriate secondary antibodies at a 1∶1000 dilution in PBST (donkey anti-rabbit Alexa 488). After incubation, slides were washed with PBST, mounted with ProLong Antifade kit (Molecular Probes, Eugene, OR, USA) for 20 min at room temperature. Images of tissues were obtained using a laser scanning confocal microscope (Zeiss LSM 510, Germany).

### Audiological evaluations

Mice were anesthetized with sodium pentobarbital (35 mg/kg) delivered intraperitoneally and maintained in a head-holder within an acoustically and electrically insulated and grounded test room. We used an evoked potential detection system (Smart EP 3.90, Intelligent Hearing Systems, Miami, FL, USA) to measure the thresholds of the auditory brainstem response (ABR) in mice. Click sounds as well as 8, 16, and 32 kHz tone bursts at varying intensity (from 10 to 130 dB SPL), were given to evoke ABRs of mice. The response signals were recorded with a subcutaneous needle electrode inserted ventrolaterally into the ears of the mice.

### Vestibular evaluations

Mice were subjected to a battery of vestibular evaluations, including observation of their circling behavior and head-tilting (performed at 3 weeks of age), a reaching test, a swimming test, a gripping test, and a rotorod test (all performed at 8 weeks of age). The reaching responses of mice were recorded after suspending animals by their tails and observing the reaction of their limbs and head-bobbing behaviors. Mice that extended their forelimbs and tried to reach a surface were considered normal (i.e., in terms of reaching response), whereas animals that either clasped their forelimbs or exhibited head-bobbing behavior were classified as abnormal. For the swimming test, mice were observed for 15–20 s, and those that maintained themselves well at the water surface were classified as normal, whereas those that failed to stay near the surface were considered abnormal. For the gripping test, mice were placed on the lower end of a 45-cm-long metal stick, and those that required more than 15 s to reach the top of the stick were classified as abnormal (i.e., <15 s were normal). For the rotorod test, mice were assessed for their ability to balance on a revolving rod (i.e., rotorod) of 3.5 cm diameter. For each test, the mouse was placed on the rod rotating at 50 rpm, and the time required for the mouse to fall was recorded. Each mouse was tested 5 times, and the results were averaged.

### Inner ear morphology studies

Tissues from inner ears of *Slc26a4^+/+^*, *Slc26a4^+/tm1Dontuh^* and *Slc26a4^tm1Dontuh/tm1Dontuh^* mice were subjected to hematoxylin and eosin (H&E) staining, and the morphology of each sample was examined with a Leica optical microscope. For light microscopy, transmission electron microscopy (TEM) and scanning electron microscopy (SEM) studies, inner ears from adult mice were fixed by perilymphatic perfusion with 4% paraformaldehyde (PFA) in phosphate-buffered saline (PBS) through round and oval windows and a small fenestra in the apex of the cochlear bony capsule. Specimens were subsequently rinsed in PBS buffer and decalcified in 4% PFA with 0.35 M EDTA at 4°C for 1 week. For light microscopy studies, samples were dehydrated and embedded in paraffin. Subsequently, serial sections (7 µm) were stained with H&E. For TEM studies, samples were post-fixed for 2 h in 2% OsO_4_ in 0.1 M PBS, dehydrated through graded ethanol solutions, and embedded in Epon. Ultrathin sections (70 nm thickness) were collected on copper grids, doubly stained with uranyl acetate and lead citrate, and examined with a Hitachi 7100 electron microscope (Hitachi, Tokyo, Japan). For SEM studies, samples were dehydrated in ethanol, critical point dried, gold sputter coated, and then examined in a field emission scanning electron microscope (S-4500; Hitachi, Tokyo, Japan).

Whole-mount studies of mouse inner ear specimens were performed as previously described [36], with some minor modification. Briefly, after perfusion with 4% PFA, the cochleae were postfixed in the same solution for 2 h at room temperature and washed in PBS. Segments of stria vascularis and organ of Corti together with Reissner's membrane were dissected out of the inner ear specimens using a fine needle. Samples were permeabilized in 1% Triton X-100 for 30 min and washed with PBS, followed by overnight incubation at 4°C in blocking solution. The tissues were then stained with rhodamine-phalloidin (1∶100 dilution; Molecular Probes, Eugene, OR, USA). After washing in PBS, the tissues were mounted using the ProLong Antifade kit (Molecular Probes, Eugene, OR, USA) for 20 min at room temperature. Images of tissues were obtained using a laser scanning confocal microscope (Zeiss LSM 510, Germany).

### Thyroid and renal serum biochemistry

Terminal blood samples were obtained from mice at 2 months and 6 months. Total T_4_ was measured in undiluted serum (25 µl) by RIA (Diagnostic Products Corp., Los Angeles, CA). Blood urea nitrogen (BUN) and serum creatinine (CREA) were measured by research services of National Taiwan University Hospital.

### Data analyses

Data are presented as mean ± SD, unless indicated otherwise. Statistical analyses included the *χ*2 or Fisher's exact test for categorical variables and Student's *t*-tests or ANOVA for continuous variables. A p value of <0.05 was considered statistically significant. All analyses were performed using SPSS/Windows software 15.0 (SPSS Science, Chicago, IL, USA).

## Supporting Information

Figure S1 ***Slc26a4^tm1Dontuh/tm1Dontuh^*** **mice revealed normal cochlear hair cells at P7.** IHC, inner hair cells; OHC, outer hair cells Bar = 50 µm.
(TIF)

Figure S2 **Plasmid construct of the c.919-2A>G mutation.** A BAC clone (clone no. bMQ23G13 Geneservice™) from the 129S7/AB2.2 BAC library containing mouse *Slc26a4* genomic region was used to construct the targeting vector. The BAC was transferred into the modified *Escherichia coli* strain EL350 by electroporation. Two homology arms flanking the genomic area of *Slc26a4* to be subcloned by gap repair were PCR amplified using BAC DNA from clone bMQ23G13 as the template, digested with *Not*I and *Hin*dIII (5′ arm) or *HindIII* and *Spe*I (3′ arm) and three-way ligated into *Not*I- and *Spe*I-digested PL253. The primers used for amplification of the 5′ and 3′ homology arms were as follows: 5′ arm F, 5′-ATAGCGGCCGCTTAAATGCTGTTTTCTCCATA-3′; 5′ arm R, 5′-AGCAAGCTTAGTCTTAAGTTGGCAGGGATGGTGAGTG-3′; 3′ arm F, 5′-TATAAGCTTAAGAGCAGCCAGTGCTCTTAACC-3′; and 3′ arm R, 5′-ATTACTAGTGTTTGGGCCTGAAGTGTAACAGC-3′. This retrieval vector was linearized by *Afl*II and *Hin*dIII digestion, gel purified and transformed into heat-shocked and electrocompetent EL350 cells containing the bMQ23G13 BAC clone. The genomic 12.47-kb region was modified in the next targeting round by inserting the neomycin (*neo*) cassette from PL451 and creating the c.919-2A>G mutation (blue asterisk) in intron 7. A *neo*/kanamycin cassette (from PL451) containing homology to a region between intron 6 and exon 8 of *Slc26a4* was inserted. The primers used to amplify the 5′ homology arm were as follows: targeting 5′ arm F, 5′-ATGGTCGACTTTCCTTGAGCTTGTTAATCTGC-3′ and targeting 5′ arm R, 5′-ATAGCGGCCGCTAGGTGCCATTTTGTGGGGTTTTG-3′. The primers used to amplify the 3′ c.919-2A>G mutation homology arm were as follows: targeting 3′ arm F, 5′-AGCGAATTCATCTTACCAGGACAATTTTTAGGATTTTTCTTTTTGATAAGACTCACATGTGCTGTTTGATGTGATATGACTTTTCCTGTGGGAGAATTG-3′; targeting 3′ arm R, 5′-ATAGCGGCCGCAAAATATATATGAGAAGATGGCTT-3′; and mutation R, 5′-ATAGGTACCACCCACTTGGGATGGACTTAACAATGCCAGCATTGTAGTTCTTTTCCAAGTTGG-3′. Homology arms were digested with *Sal*I and *Bam*HI (5′ arm) and *Eco*RI and *Not*I (3′ arm), and four-way ligated with the *Eco*RI- and *Bam*HI-digested *neo* cassette from PL451 and *Sal*I- and *Not*I-digested PL451 backbone. The targeting cassette was released by *Sal*I and *Not*I digestion and inserted into the retrieved *Slc26a4* fragment by recombineering.
(TIF)
