# Supplementary figures and images for: Establishment of a Knock-In Mouse Model with the SLC26A4 c.919-2A>G Mutation and Characterization of Its Pathology
Source: PLoS One. 2011 Jul 21;6(7):e22150. doi: 10.1371/journal.pone.0022150 (PMC3141011; doi:10.1371/journal.pone.0022150)

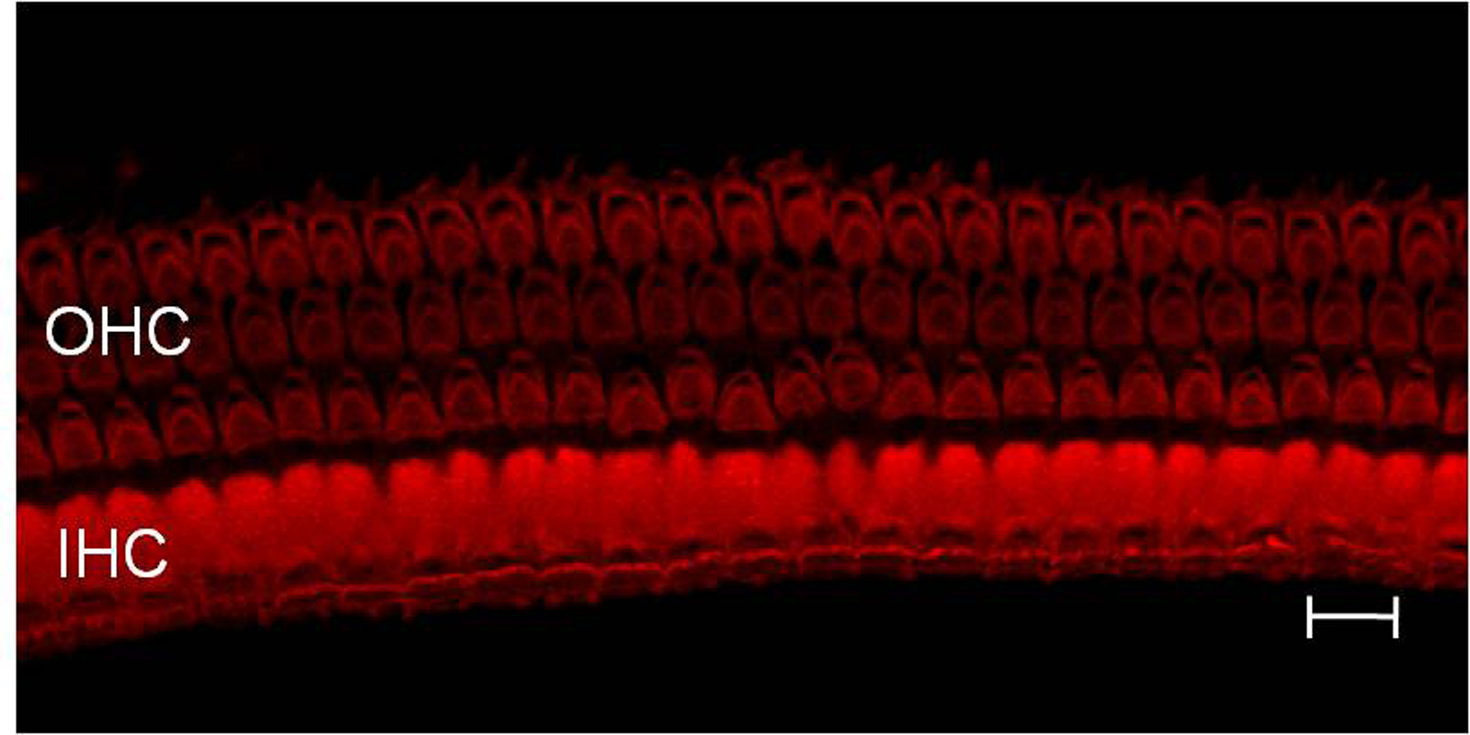

Supplement: Figure S1 — Slc26a4tm1Dontuh/tm1Dontuh mice revealed normal cochlear hair cells at P7. IHC, inner hair cells; OHC, outer hair cells Bar = 50 µm. (TIF) [file pone.0022150.s001.tif]

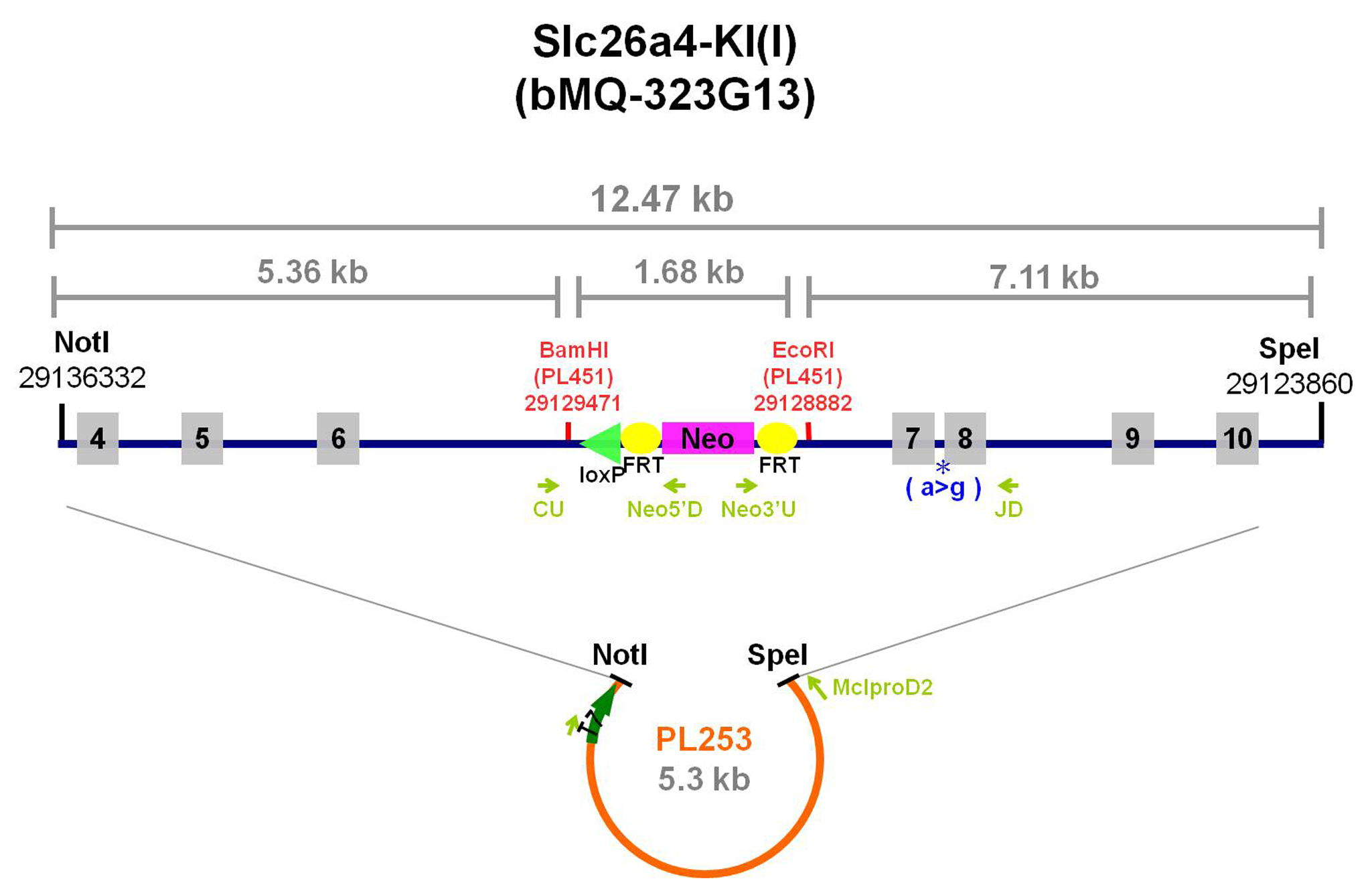

Supplement: Figure S2 — Plasmid construct of the c.919-2A>G mutation. A BAC clone (clone no. bMQ23G13 Geneservice™) from the 129S7/AB2.2 BAC library containing mouse Slc26a4 genomic region was used to construct the targeting vector. The BAC was transferred into the modified Escherichia coli strain EL350 by electroporation. Two homology arms flanking the genomic area of Slc26a4 to be subcloned by gap repair were PCR amplified using BAC DNA from clone bMQ23G13 as the template, digested with NotI and HindIII (5′ arm) or HindIII and SpeI (3′ arm) and three-way ligated into NotI- and SpeI-digested PL253. The primers used for amplification of the 5′ and 3′ homology arms were as follows: 5′ arm F, 5′-ATAGCGGCCGCTTAAATGCTGTTTTCTCCATA-3′; 5′ arm R, 5′-AGCAAGCTTAGTCTTAAGTTGGCAGGGATGGTGAGTG-3′; 3′ arm F, 5′-TATAAGCTTAAGAGCAGCCAGTGCTCTTAACC-3′; and 3′ arm R, 5′-ATTACTAGTGTTTGGGCCTGAAGTGTAACAGC-3′. This retrieval vector was linearized by AflII and HindIII digestion, gel purified and transformed into heat-shocked and electrocompetent EL350 cells containing the bMQ23G13 BAC clone. The genomic 12.47-kb region was modified in the next targeting round by inserting the neomycin (neo) cassette from PL451 and creating the c.919-2A>G mutation (blue asterisk) in intron 7. A neo/kanamycin cassette (from PL451) containing homology to a region between intron 6 and exon 8 of Slc26a4 was inserted. The primers used to amplify the 5′ homology arm were as follows: targeting 5′ arm F, 5′-ATGGTCGACTTTCCTTGAGCTTGTTAATCTGC-3′ and targeting 5′ arm R, 5′-ATAGCGGCCGCTAGGTGCCATTTTGTGGGGTTTTG-3′. The primers used to amplify the 3′ c.919-2A>G mutation homology arm were as follows: targeting 3′ arm F, 5′-AGCGAATTCATCTTACCAGGACAATTTTTAGGATTTTTCTTTTTGATAAGACTCACATGTGCTGTTTGATGTGATATGACTTTTCCTGTGGGAGAATTG-3′; targeting 3′ arm R, 5′-ATAGCGGCCGCAAAATATATATGAGAAGATGGCTT-3′; and mutation R, 5′-ATAGGTACCACCCACTTGGGATGGACTTAACAATGCCAGCATTGTAGTTCTTTTCCAAGTTGG-3′. Homology arms were digested with SalI and BamHI (5′ arm) and EcoRI and NotI (3 [file pone.0022150.s002.tif]
